# Broadband Air-Coupled Ultrasound Emitter and Receiver Enable Simultaneous Measurement of Thickness and Speed of Sound in Solids

**DOI:** 10.3390/s23031379

**Published:** 2023-01-26

**Authors:** Klaas Bente, Janez Rus, Hubert Mooshofer, Mate Gaal, Christian Ulrich Grosse

**Affiliations:** 1 Bundesanstalt für Materialforschung und -prüfung BAM, 8.4 Acoustic and Electromagnetic Methods, Unter den Eichen 87, 12205 Berlin, Germany; 2 Health and Medical University, Olympischer Weg 1, 14471 Potsdam, Germany; 3 Chair of Non-Destructive Testing, Centre for Building Materials, Technical University of Munich, Franz-Langinger-Straße 10, 81245 Munich, Germany; 4 Laboratory of Wave Engineering, Swiss Federal Institute of Technology Lausanne, Station 11, 1015 Lausanne, Switzerland; 5 Siemens AG, Corporate Technology, Otto-Hahn-Ring 6, 81739 München, Germany

**Keywords:** thermoacoustic emitter, optical microphone, air-coupled ultrasound, local resonance, thickness measurement, thickness resonance

## Abstract

Air-coupled ultrasound sensors have advantages over contact ultrasound sensors when a sample should not become contaminated or influenced by the couplant or the measurement has to be a fast and automated inline process. Thereby, air-coupled transducers must emit high-energy pulses due to the low air-to-solid power transmission ratios (10^−3^ to 10^−8^). Currently used resonant transducers trade bandwidth—a prerequisite for material parameter analysis—against pulse energy. Here we show that a combination of a non-resonant ultrasound emitter and a non-resonant detector enables the generation and detection of pulses that are both high in amplitude (130 dB) and bandwidth (2 µs pulse width). We further show an initial application: the detection of reflections inside of a carbon fiber reinforced plastic plate with thicknesses between 1.7 mm and 10 mm. As the sensors work contact-free, the time of flight and the period of the in-plate reflections are independent parameters. Hence, a variation of ultrasound velocity is distinguishable from a variation of plate thickness and both properties are determined simultaneously. The sensor combination is likely to find numerous industrial applications necessitating high automation capacity and opens possibilities for air-coupled, single-side ultrasonic inspection.

## 1. Introduction

Most currently used air-coupled ultrasound (ACU) transducers can be classified as either piezoelectric or capacitive in nature [1,2,3,4,5]. However, developments in recent years have resulted in fundamentally new approaches to generate and detect ultrasound in air [6]. Thermoacoustic emitters were proven to provide high amplitude and high-bandwidth acoustic signals with frequencies of up to 1 MHz [7]. At the same time, highly sensitive optical microphones were developed that cover the same frequency range [8,9,10,11]. This new generation of emitters and receivers can overcome a former fundamental restriction of air-coupled ultrasound: only transducers with pulse durations above 10 µs provided sufficient amplitude for practical applications. Previously proposed methods to generate and detect pulses shorter than 10 µs resulted in low amplitudes [1,12]. However, high amplitudes are mandatory for air-coupled ultrasound applications due to the low energy transmission coefficients at the involved solid-to-air interfaces.

Here we demonstrate the combined use of a broadband, thermoacoustic emitter and an optical microphone and show an initial application: the simultaneous determination of thickness and sound velocity of a solid material. Carbon fiber-reinforced plastic (CFRP) plates with different thicknesses were used to show the applicability for a currently relevant material [13]. A transmission setup and time-of-flight data processing were implemented.

Previously proposed techniques that measure thickness and speed of sound simultaneously rely on the thickness resonances (TR) of the investigated sample [5]. Such techniques, however, require the determination of the frequency-dependent transmission coefficient, necessitating some advanced data processing and data fitting. More importantly, the correct transducer combination is required for each new sample. The sensor combination presented here is simpler to use, and the transducer pair works for all samples that have a TR frequency below a critical value of 1 MHz.

A plate thickness measurement has been demonstrated previously using a laser pulse to generate the ultrasound and an optical microphone as a detector [14]. Since the ultrasound was generated directly in the specimen, the distance between the source of the ultrasound and the detector was not constant during the scan. It was thus not possible to extract the plate thickness independently from the plate material (and vice versa), as in the case for our method. In [14], the plate thickness and plate curvature were measured, while the corresponding sound velocity in the plate needed to be known or measured at the reference point. This is not necessary for the method introduced in this work.

## 2. Materials and Methods

### 2.1. Transducers

**Figure 1:**
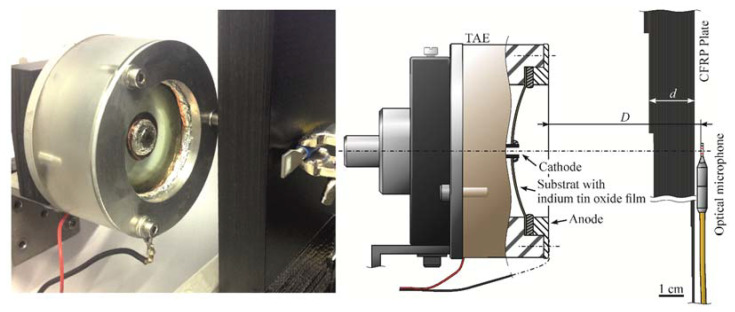
Image and schematic of the air-coupled ultrasound transmission setup. Sending and receiving transducers were positioned on both sides of the CFRP step wedge. The emitter consists of a thin indium tin oxide film in between two electric poles. The receiver was an optical microphone. The distance between transmitter and receiver *D* and the thickness of the plate could be determined experimentally.

The thermoacoustic effect describes the generation of sound from heat. Well-known cases are thunder and spark discharge [15], but also less commonly known cases such as laser-induced breakdown [16] and thermophones [7,17,18] are capable of transforming heat into acoustic waves. Physically best understood are thermophones, which use a thin Ohmic conductor to transform electric energy into heat in the surrounding fluid. When the heat deposition occurs fast enough, a finite volume of air is heated, which is equivalent to an increase in pressure. Thin films on a curved surface are well suited for material testing [7]. Such setups hold the advantages of a thermoacoustically active 2D area that can be used for pressure generation and beam focusing at the same time. Typical materials are indium tin oxide for the thin film and silica glass for the substrate. The transducer used in this study consisted of a 200 nm indium tin oxide film on a borosilicate glass substrate with a curvature of 55 mm and radial electrode placement (see Figure 1). Thermoacoustic transducers generate ultrasound directly in air and do not rely on mechanical or resonant vibrations. This enables the generation of short pulses, necessary for the method proposed in this work.

The receiver was an optical microphone (Eta 450 Ultra, XARION Laser Acoustics GmbH, Vienna, Austria). The working principle is based on a rigid Fabry-Pérot laser interferometer with two miniaturized mirrors, where sound waves in air change the refractive index, alter the optical wavelength and the light transmission of the pair of mirrors. Like the thermoacoustic emitter, the microphone does not rely on resonantly vibrating components to detect ultrasound and hence enables broadband signal detection.

**Figure 2:**
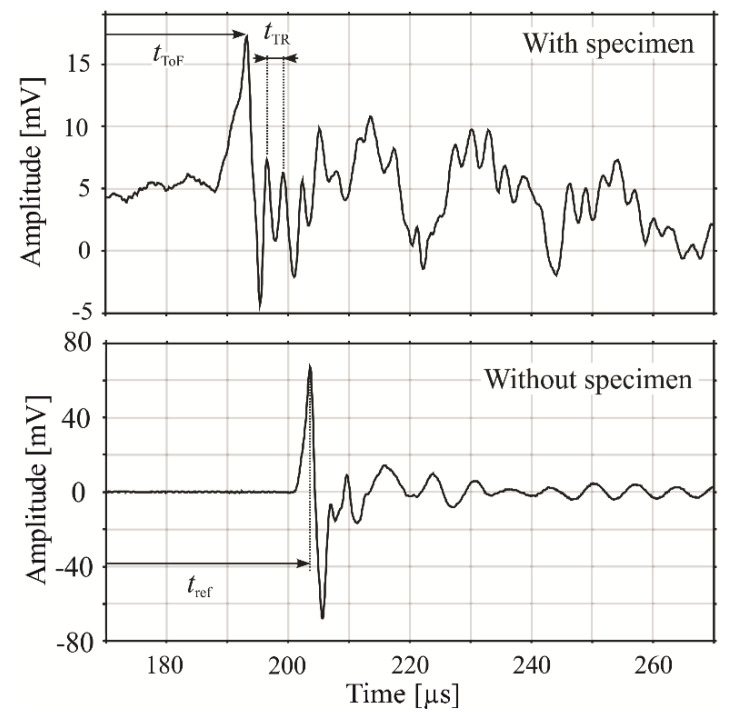
Representative signals for the proposed technique. The upper and lower graphs show a measured signal with and without a specimen, respectively. The times $t_{\mathrm{TR}}$ and $t_{\mathrm{ToF}}$ are independent parameters, which enable the discrimination between the influence of the plate thickness and the material properties. An initial measurement of the time of flight between the transducers ($t_{\mathrm{ref}}$) enables the determination of absolute values for both parameters simultaneously. The observable signal oscillations after the pulse detection feature components other than only the in-plate reflections and are further analyzed in the signal measurement and processing section.

It is the combination of a high-bandwidth transmitter and a high-bandwidth receiver that lets us resolve the in-plate reflections in time even for millimeter thin composite plates, as discussed later in this article. The measured signal of the setup with and without a specimen is shown in Figure 2.

### 2.2. Calculation of Thickness and Speed of Sound

High-power microsecond pulses in CFRP plates enable exact determination of the pulse arrival time $t_{\mathrm{ToF}}$ and the simultaneous measurement of the in-plate reflection period $t_{\mathrm{TR}}$, as shown in Figure 2. This enables the determination of plate thickness and longitudinal ultrasound velocity in the investigated plate from a single measurement.

We show this by expanding on the time-of-flight calculations from [19], in which only one of the two parameters could be determined independently. In addition, note that reference [19] reports a speed of sound measurements with uncertainties mostly in the range of 20–50% due to long pulse widths.

In the following, $t_{\mathrm{ToF}}$ is the time the pulse travels through air and sample, *D* is the corresponding distance between ultrasound source and detector, *d* is the material thickness, and $v_{A}$ and $v_{M}$ are the speed of sound in air and in the analyzed material, respectively. With these definitions, we can express $t_{TR}$ and $t_{\mathrm{ToF}}$ as

(1) $$t_{TR}=\frac{2d}{v_{M}}$$

and

(2) $$t_{\mathrm{ToF}}=\frac{d}{v_{M}}+\frac{D-d}{v_{A}}.$$

These two equations can be solved for the plate thickness *d* and the longitudinal ultrasound velocity in the plate material $v_{M}$. Setting up two transducers with a distance *D* is typically error prone with uncertainties in the order of magnitude of 1 mm. This leads to a similar error in the calculation of *d*. Therefore, it is best practice to replace the measurement of *D* by a measurement of the time of flight of a pulse between the two transducers with no sample $t_{\mathrm{ref}}$, meaning $D=t_{\mathrm{ref}}\cdot v_{A}$. These considerations lead to

(3) $$d=\left(t_{\mathrm{ref}}-t_{\mathrm{ToF}}+\frac{t_{\mathrm{TR}}}{2}\right)\cdot v_{A}$$

and

(4) $$v_{M}=\frac{2d}{t_{\mathrm{TR}}}.$$

Both parameters, *d* and $v_{M}$, can be estimated from the independent parameters $t_{\mathrm{ToF}}$ and $t_{\mathrm{TR}}$, obtained from a single ultrasonic signal. The only prerequisites for this obtainment are the detectability of the in-plate reflections and the constant distance between the transducers.

### 2.3. Experimental Setup

The air-coupled ultrasound transmission setup can be divided into four parts: the sending part, the receiving part, the manipulator, and the piloting computer. The sending part consisted of the USPC 4000 Airtech system (Hillger NDT GmbH, Braunschweig, Germany), a voltage divider, an Agilent 33500B (Keysight Technologies, Santa Rosa, CA, USA) arbitrary waveform generator (AWG), an in-house power amplifier and the already described thermoacoustic transducer. The USPC 4000 Airtech system generated electric trigger signals, which were adjusted to 5 V and transmitted to the AWG. The pulse width was adjusted at the AWG and the pulse was transmitted to the in-house power amplifier and transmitted to the thermoacoustic transducer.

The power amplifier was designed such that pulses in the order of magnitude of several 100 ns to several 100 µs and 30 kW could be generated for a thermoacoustic load with a resistance of 8 Ω.

The receiving part consisted of the optical microphone, its data processing unit, and an analog-to-digital converter, which was part of the USPC 4000 Airtech system. The manipulator was a FlatScan 1000 (Hillger NDT GmbH, Braunschweig, Germany), controlled by the USPC 4000 Airtech system. Hillgus software (Hillger NDT GmbH, Braunschweig, Germany) was used to synchronize the sent and received signals and to control the manipulator position.

The transducers were facing one another, and their distance was varied until a maximum amplitude could be measured on the receiving side. This distance was 54 mm for all thicknesses. The pulse width of the electric pulse was set to 2 µs at an amplitude of 375 V. Together with the 7.8 Ohm of the transducer, this resulted in an approximately 18 kW electrical sending power. The pulses were transmitted at a 75 Hz repetition rate and the sample was scanned with a spatial resolution of 0.15 mm along the scanning axis. Three adjacent lines with 0.15 mm distance were scanned and the results averaged. This avoided artefacts caused by local sample inhomogeneities. The signal converter of the optical microphone was set to 20 dB amplification and the data acquisition software Hillgus amplified the signal by another 9 dB for all performed scans. The temperature was 23 °C and the relative humidity was 40%.

### 2.4. Specimen

The step wedge was a CFRP made from an epoxy prepeg of type HexPly(R). The fiber density was 1.78 g/cm^3^ and the resin density 1.22 g/cm^3^. The fiber orientation was quasi isotropic with 120 layers over a total thickness of 20.2 mm.

The specimen features a surface roughness below the smallest wavelength components of sound in both interface media, parallel front and back surfaces, and a sufficiently low dispersion. These parameters are typical prerequisites for air-coupled ultrasonic testing.

## 3. Results

### 3.1. Signal Measurement and Processing

**Figure 3:**
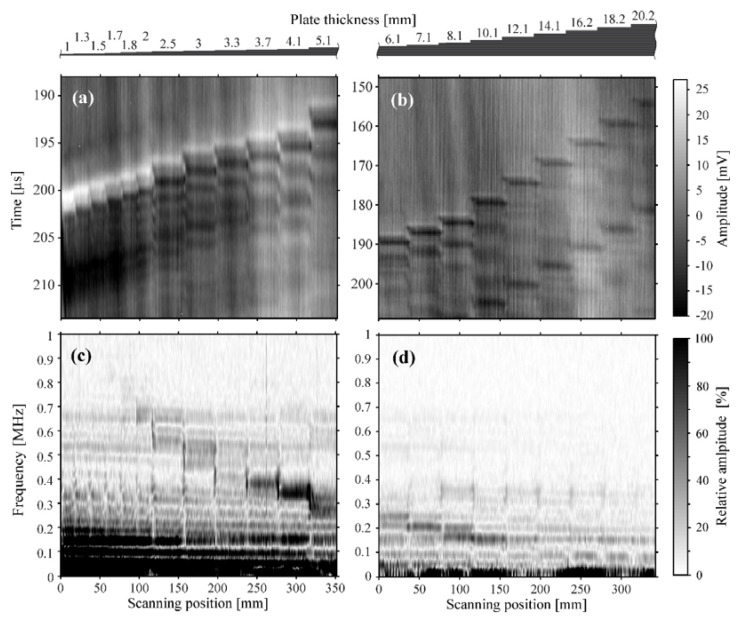
B-scans for thinner (**a**) and thicker (**b**) plate thickness ranges. The signals in frequency domain are shown in S-scans (B-scans in frequency domain) for the same positions of both linear scans in (**c**,**d**). The TR frequency is inversely proportional to the plate thickness, while the ToF is linearly proportional to the plate thickness.

Linear scans were performed using plates with thicknesses ranging from 1 mm to 5.1 mm (Figure 3a) and from 6.1 mm to 20.2 mm (Figure 3b), as indicated above the figures. Figure 3a,b have different time windows since the trigger time delays were not the same for both scans. The delays were chosen such that the first break and the subsequent in-plate reflections are fully captured for all plate thicknesses.

The obtained signals were converted to frequency domain for all scanning positions. The linear scans of the plate thicknesses are shown in S-(spectrum) scans (Figure 3c,d). The term S-scan is used to designate B-scans in frequency domain. In order to improve the signal-to-noise ratio, only the time window between 10 µs before and 32 µs after the first break of the signal was used. It was converted to frequency domain, after applying a Hamming window. The amplitude of the S-scans is expressed relative to the maximum peak value of the TR frequency of both linear scans.

The goal of the signal processing is to determine $t_{\mathrm{ToF}}$ and $t_{\mathrm{TR}}$ in an automated process. The value of $t_{\mathrm{TR}}$ is determined in frequency space. The in-plate reflections and the TR frequency are distinguishable for the plate thicknesses ranging from 1.7 mm to 10.1 mm. Figure 3c,d clearly show that the TR frequency peak value is proportional to $d^{-1}$. Although our system is comparatively broadband, it is to some extent possible to observe its characteristic behavior. Each signal was superimposed by two main oscillations with period times of roughly 10 µs and 30 µs (see Figure 2). These signals were likely caused by multiple reflections of the pulse inside the microphone cavity and between the microphone and the plate surface, respectively. Such interferences leads to differences in the quality of the signal for certain plate thicknesses. For example, the detected TR frequencies close to 660 kHz, 540 kHz, 340 kHz, and 150 kHz have higher amplitude than others. However, despite these interferences, the method allows for reliable detection of the TR frequency peak over a broad frequency range.

The value of $t_{\mathrm{ToF}}$ was determined in time domain. A strong correlation between the plate thickness and $t_{\mathrm{ToF}}$ is shown in Figure 3a,b. The in-air reflections between the specimen and the optical microphone are visible in Figure 3b. They arrive approximately 30 µs after the first break of the US signal. This corresponds to the microphone-to-specimen distance of 4.8 mm.

### 3.2. Comparison of Measured and Reference Values

The first step of the analysis was the extraction of $t_{\mathrm{ToF}}$ and $t_{\mathrm{TR}}$ from the signal for all scanning positions. $t_{\mathrm{ToF}}$ was reliably obtained by picking the time of the maximum signal levels (shown in Figure 3a) for the scan over the lower plate thickness range (1 mm to 5.1 mm). For greater thicknesses (6.1 mm to 20.2 mm), the first negative peak is more pronounced than the first positive peak (Figure 3b). The arrival times were thus more reliably obtained by picking the time of the minimum signal level. The in-air reflections, which arrived after the first break of the signal and might have larger amplitude, were neglected. The mean value of the arrival time difference between the minimum and the maximum signal values of all the scanning locations was added to the arrival times for the scan of the lower thickness range in order to synchronize the scans of both thickness ranges.

The parameter $t_{\mathrm{ToF}}$ was obtainable by this method for all plate thicknesses. This was not the case for the second parameter $t_{\mathrm{TR}}$, which was not measurable for the whole thickness range. As can be seen in Figure 3, the in-plate reflections (in time domain) and TR peak (frequency domain) are visible only for the plate thicknesses between 1.7 mm and 10.1 mm. This is due to the frequency range of our measurement system—up to 0.8 MHz for the applied excitation parameters. The upper limit of the plate thickness is defined by the ultrasound attenuation level in the plate. The condition for the successful measurement is that the in-plate reflections are detectable. $t_{\mathrm{TR}}$ was obtained by inverting the TR peak (visible in Figure 3c,d).

**Figure 4:**
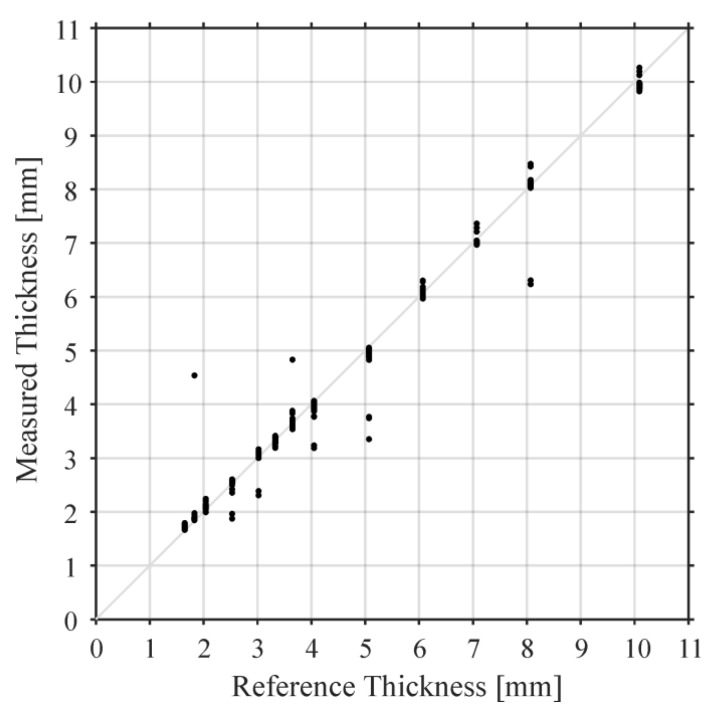
Plate thicknesses obtained by simultaneous measurement of $t_{\mathrm{TR}}$ and $t_{\mathrm{ToF}}$ using Equation (3). As $t_{\mathrm{TR}}$ and $t_{\mathrm{ToF}}$ are independent parameters, we can simultaneously estimate longitudinal sound velocity in the sample material (approximately 2800 m/s) using Equation (4), without a reference measurement at a known thickness, which is required for conventional contact ultrasonic measurements. This is only possible because our method is broadband and air coupled.

In Figure 4, we present the plate thicknesses obtained by inserting $t_{\mathrm{ToF}}$ and $t_{\mathrm{TR}}$ in Equations (3) and (4). The only parameter in this equation that was not measured directly by our experiment is sound velocity in air which proportionally influences all the measurement points and amounted to 345.7 m/s in our experiment. The thicknesses measured by our ACU method are compared to values obtained by the reference measurement with a micrometer screw (precision higher than 0.01 mm). In Table 1, statistical values are provided for each of the plate thicknesses: mean value of the air-coupled measurement, its standard deviation, number of measurements at each of the plate thicknesses (obtained by the linear scan), and the number of the outliers, which we excluded to calculate the standard deviation. We did not exclude any values in Figure 4.

The outliers are caused by the false picking of the time of arrival or the TR peak frequency. The two parameters might not refer to the same thickness, especially at the (discrete) transition from one thickness to another. The wave propagation properties could be affected by the thickness transitions, which could alter the TR frequency (Figure 3). The standard deviations of the air-coupled thickness measurements remained below 0.09 mm for the plate thicknesses from 1.65 mm and 10.1 mm.

The longitudinal sound velocity in the plate material was measured for all scanning locations by the same experiment using Equation (4). Its mean value over the whole scan is 2800 m/s with the standard deviation of 160 m/s. A reference measurement of the speed of sound in the utilized plate was performed using a contact ultrasound time of flight measurement with a 2.25 MHz transducer. The technique produced 2896 m/s, which is well within the first standard deviation of the proposed air-coupled technique.

## 4. Discussion

Our experimental setup enables, for the first time, the resolution of in-plate reflections at plate thicknesses ranging from 1.65 mm to 10 mm without physical contact to the specimen. In contrast to conventional pulse echo methods based on contact ultrasound, the time of flight and the period of in-plate reflections are, in our setup, independent parameters. This opens the possibility to measure the speed of sound and material thickness simultaneously from a single ultrasonic signal. In other words, it is possible to measure plate thickness without actually knowing the ultrasound propagation speed in the sample material. This new paradigm only assumes the prior knowledge of the speed of sound in air and a reference time-of-flight measurement without the specimen. The latter will deliver the distance between the transducers, which should and can (due to the air-coupled setup) be kept constant during the whole experiment.

The experiments were conducted at a CFRP plate due to its relevance in research and industry. The applicability of our technique to a certain object will depend on the object’s composition of speed of sound, thickness, and dispersion. Dispersion might influence our technique stronger than other material analysis approaches as it may vary the shape of the in-plate reflections. Applying our technique to a wide range of material parameter combinations will be a subject of future research.

Temperature changes and airflows are likely to occur in industrial applications. The airflows can influence air-coupled ultrasound measurements when the overall distance between the sender and the receiver in air is several centimetres long. Such flows might disrupt the method by altering the measured time of flight in air. It is, however, unlikely that temperature changes will influence our measurement since they occur on different time scales than the ones relevant for our method—200 µs.

We measured signal components up to 0.8 MHz. However, the non-resonant working principles of the utilized emitter and receiver do not limit this frequency range. A limiting factor is the energy per pulse that is proportional to the pulse length and amplitude. The pulse width (e.g., full width at half maximum, $\tau_{\mathrm{FWHM}}$), however, determines the lower limit of detectable plate thicknesses $d_{\mathrm{limit}}$ via $d_{\mathrm{limit}}=c\cdot \tau_{\mathrm{FWHM}}/2$. Higher amplitudes will be required for future applications that require even shorter pulse lengths. With further developments in the efficiency of thermoacoustic emitters and the sensitivity of optical receivers, sub-microsecond pulses are likely to be provided for future applications.

The proposed technique is one initial possibility for the utilization of short pulses and non-resonant transducers and more applications are likely to follow. As our method is able to distinguish the in-plate reflections, it provides the potential to allow single-side air-coupled ultrasonic inspection and to resolve the direct reflection from the back-wall echo. Localized material properties of the specimen or the inspected features could be characterized by the analysis of the local resonances in the ultrasonic frequency range, as it has been demonstrated using laser generated ultrasound [15,20]. The broadband thermoacoustic emitter carries high potential to replace laser pulse ultrasound excitation [21], which is more expensive and evokes special safety-related concerns. As both transducers also function in liquids, expanding the scope of the technique to immersion testing is likely to succeed in future research.

## Figures and Tables

**Table 1:** Plate thicknesses determined with the presented ultrasound method with reference values.

Reference [mm]	Air-Coupled—Mean Value [mm]	Air-Coupled—SD ^1^ [mm]	Number of Measurements	Number of Excluded Outliers
1.65	1.70	0.03	23	0
1.83	1.89	0.03	22	1
2.04	2.11	0.05	22	0
2.53	2.55	0.05	45	2
3.02	3.06	0.04	43	2
3.33	3.32	0.04	44	0
3.65	3.62	0.07	46	1
4.05	3.99	0.06	46	2
5.07	4.96	0.06	38	3
6.07	6.07	0.08	43	0
7.07	7.03	0.07	44	0
8.07	8.12	0.08	45	2
10.90	9.93	0.08	46	0

^1^ SD = standard deviation.

## Data Availability

Not applicable.
